# The Risk of Septicemia in End-Stage Renal Disease With and Without Renal Transplantation: A Propensity-Matched Cohort Study

**DOI:** 10.1097/MD.0000000000001437

**Published:** 2015-08-28

**Authors:** Te-Chun Shen, I-Kuan Wang, Chang-Ching Wei, Cheng-Li Lin, Chia-Ta Tsai, Te-Chun Hsia, Fung-Chang Sung, Chia-Hung Kao

**Affiliations:** From Graduate Institute of Clinical Medicine Science, College of Medicine, China Medical University, Taichung, Taiwan (T-CS, I-KW, C-TT, F-CS, C-HK); Division of Pulmonary and Critical Care Medicine, Department of Internal Medicine, China Medical University Hospital, Taichung, Taiwan (T-CS, T-CH); Intensive Care Unit, Chu Shang Show Chwan Hospital, Nantou, Taiwan (T-CS); Division of Nephrology, Department of Internal Medicine, China Medical University Hospital, Taichung, Taiwan (I-KW); Division of Nephrology, Department of Pediatrics, China Medical University Hospital, Taichung, Taiwan (C-CW); Management Office for Health Data, China Medical University Hospital, Taichung, Taiwan (C-LL, F-CS); Division of Infection, Department of Internal Medicine, China Medical University Hospital, Taichung, Taiwan (C-TT); and Department of Nuclear Medicine and PET Center, China Medical University Hospital, Taichung, Taiwan (C-HK).

## Abstract

End-stage renal disease (ESRD) is a well-known risk factor for septicemia. Renal transplantation (RTx) is the treatment of choice for ESRD. However, RTx recipients should undergo long-term immunosuppressive therapy. The aim of this study was to evaluate the risk of septicemia in ESRD patients with and without RTx.

This cohort study used the National Health Insurance (NHI) data of Taiwan from 2000 to 2010. The RTx group consisted of 3286 RTx recipients. The non-RTx comparison group also consisted of 3286 subjects with ESRD matched by propensity scores for age, sex, index date, comorbidities, and medications. The subjects were followed until the end of 2011 to evaluate the septicemia risk.

The risk of septicemia was lower in the RTx group than the non-RTx group, with an adjusted hazard ratio of 0.73 [95% confidence interval (CI) = 0.64–0.84, *P* < 0.001]. In addition, we observed insignificantly lower intensive care unit (ICU) admission rate (35.8% vs. 39.8%) and lower 30-day all-cause mortality rate (17.2% vs. 18.5%) in the RTx group than the non-RTx group. However, the mean cost for septicemia in the RTx group was insignificantly higher than the non-RTx group (7175 vs. 6421 USD, *P* = 0.39).

RTx recipients had a significantly reduced risk of developing septicemia compared to the propensity-matched non-RTx ESRD patients. The ICU admission and 30-day all-cause mortality rates also slightly decreased in RTx recipients but without statistical significance.

## INTRODUCTION

Patients with end-stage renal disease (ESRD) are at a higher risk of acquiring infections than the general populations.^1^ Infection is the second highest cause of mortality following cardiovascular diseases in ESRD patients. Septicemia is one of the most severe type of infection in ESRD patients.^2^ The mortality rate in uremic patients with septicemia was between 12% and 22%.^3^ The mechanisms through which ESRD predisposes to infection include alterations of primary host defense, advanced age, and the presence of comorbid conditions such as diabetes, invasive dialysis procedures, disruption of skin and mucosa barriers, malnutrition, and susceptibility to nosocomial transmission.^4^

Renal transplantation (RTx) is the most common form of solid organ transplant and is the treatment of choice for ESRD, because it confers a progressive survival benefit and is effective in improving quality of life of patients.^5,6^ However, patients who receive RTx require lifetime immunosuppressive therapy to avoid rejection of the transplanted allograft. Therefore, RTx recipients are exposed to pathogens in a sustained immunosuppressive state. Infection is thus the second leading cause of death in RTx recipients and it is associating with the decreased transplant survival.^7,8^

Several studies have reported on the relationship between RTx and infections. Bige et al^9^ reported that the most common sites of infection requiring intensive care unit (ICU) admission in RTx recipients include the lungs (54%), urinary tract (24%), and bloodstream (22%). Mouloudi et al^10^ reported the mortality rate was 2.4-fold higher for RTx recipients with infection requiring ICU admissions than for noninfectious RTx recipients requiring ICU admissions (62.9% vs. 26.5%). Among RTx recipients with severe sepsis and septic shock, hospital mortality was associated with male gender, worse Sequential Organ Failure Assessment (SOFA) scores, hemodynamic instability, use of mechanical ventilation, and advanced graft dysfunction.^11^

At present, the population-based cohort study comparing infection risk between ESRD patients with and without RTx is lacking. The purpose of this study was to evaluate the subsequent risk of septicemia for ESRD patients who underwent RTx and compared with well-matched ESRD patients who did not undergo RTx. We also measured the risk of ICU admission, 30-day all-cause mortality, and the costs of treatment. The data were obtained from the National Health Insurance (NHI) system of Taiwan, which provides a nationwide, large-scale cohort dataset and has been used for various studies over several years.

## MATERIALS AND METHODS

### Data Source

This study used data sets extracted from the National Health Insurance Research Database (NHIRD) of the Taiwan NHI program, in which >99% of the population of Taiwan have been enrolled (http://www.nhi.gov.tw/english/index.aspx). These data sets contained registration files and original medical claims data of all beneficiaries with encrypted unique personal identifications to secure patients’ confidentiality. All data sets were linked using unique surrogate personal identification numbers to obtain the longitudinal medical records of each insured person. Information for patients with ESRD was obtained from the registry for catastrophic illness patient database (RCIPD). All registered ESRD patients have been prescribed for the long-term renal replacement therapy for dialysis or RTx. Diagnoses were identified by the International Classification of Diseases, Ninth Revision, Clinical Modification (ICD-9-CM). This study was evaluated and approved by the Institutional Review Board of China Medical University and Hospital (CMU-REC-101-012).

### Study Subjects

From RCIPD, we identified 152,429 patients with ESRD (ICD-9-CM code 585.6) newly diagnosed in the 2000 to 2010 period. Among them, 4297 patients had received RTx. After excluding those aged < 20 years or without demographic status (n = 111), with septicemia (ICD-9-CM code 038, 790.7) (n = 406) and with graft failure (n = 259), 3521 patients were selected for the RTx group. The date of receiving RTx was defined as the index date. Comparison subjects in the non-RTx group were randomly selected from ESRD patients registering in RCIPD. To reduce selection bias, frequency matching by index date and propensity score was applied to select the 2 cohorts with and without RTx in a 1:1 ratio.^12^ The propensity score was calculated using logistic regression to estimate the probability of the RTx assignment based on the baseline variables including age, sex, index date, comorbidities (hepatitis B, hepatitis C, and Charlson Comorbidity Index [CCI] score), and medications (steroids and immunosupressants [cyclosporine, azathioprine, mycophenolate mofetil, and tacrolimus]).

**FIGURE 1:**
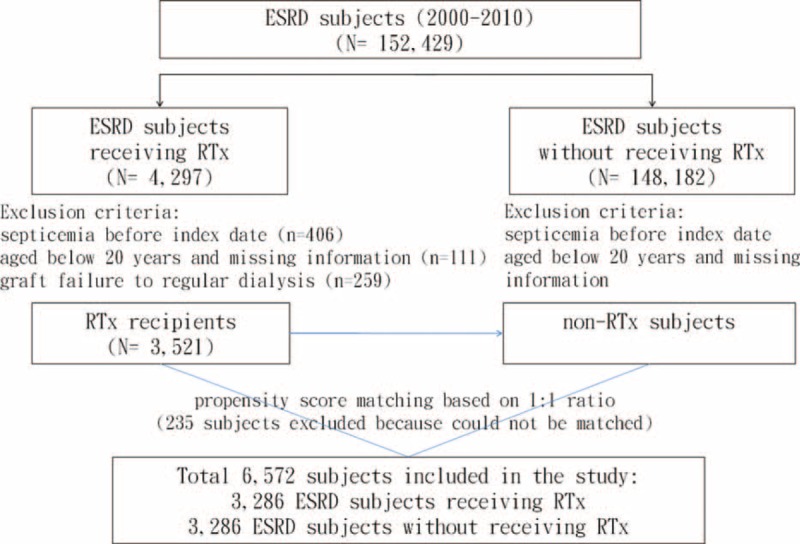
Schematic diagram for establishing study cohorts.

We used a modified method to evaluated CCI score for each patient relying on ICD-9-CM diagnosis and procedure codes.^13^ Items to be evaluated were cardiovascular diseases, cerebrovascular diseases, chronic pulmonary diseases, rheumatologic diseases, peptic ulcer diseases, liver diseases, diabetes and complications, renal diseases, malignancy, and human immunodeficiency virus infection (renal diseases were excluded in the present study). After further excluding 235 subjects could not be matched, the study cohorts consisted of 3286 subjects in RTx group (including 18 subjects with retransplantations) and 3286 subjects in non-RTx comparison group (received no RTx during the whole study period) (Figure 1). The average dialysis time before index date was 3.67 (SD = 3.30) years in the RTx group and 3.72 (SD = 5.91) years in the non-RTx comparison group. All subjects were followed-up until the occurrence of septicemia or censored because of death, withdrawal from the insurance program, or December 31, 2011 (whichever occurred first).

### Statistical Analysis

The Chi-square test was used to examine the difference in categorical variables between the 2 groups, while the 2 sample *t* test was used to examine continuous variables. We calculated the incidence densities of septicemia in both RTx and non-RTx groups. Cox proportional hazards regression model was used to measure hazard ratio (HR) of septicemia and 95% confidence interval (CI) for the RTx group compared with the non-RTx group. Moreover, logistic regression analysis was used to measure the odds ratio (OR) of ICU admission and 30-day all-cause mortality since the diagnosis of septicemia, which could be an admission date or rarely an outpatient department visiting date, compared between the 2 groups. The Cox proportional hazards model was also used to calculate the adjusted cumulative incidence of septicemia for both RTx and non-RTx groups. All statistical analyses were performed using SAS 9.3 statistical software (SAS Institute, Inc., Cary, NC). The cumulative incidence plot was drawn by using R 3.0.01.^14^ The significance level for all analyses was set to a *P*-value of 0.05.

## RESULTS

Table 1 shows that the 2 study cohorts were similar in distributions of gender CCI score, comorbidities, and steroids use at the baseline. There were more men than women. The RTx group was slightly older and more likely took immunosupressants than the non-RTx group.

**TABLE 1:**
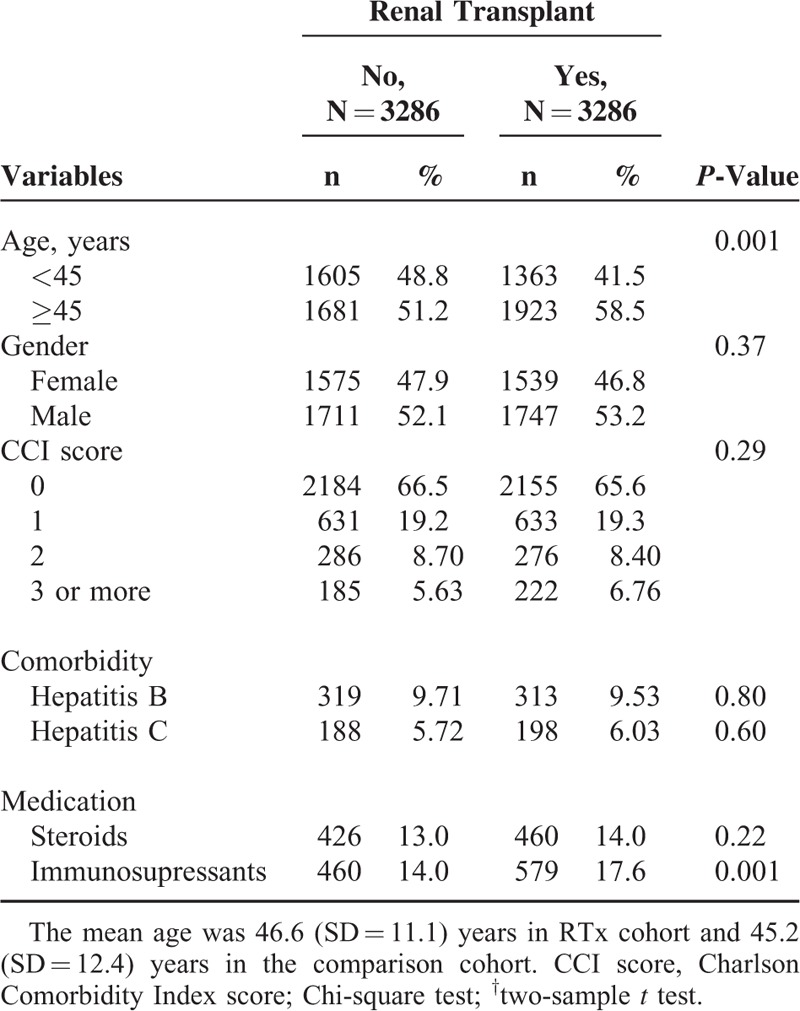
Demographic Characteristics, Comorbidity, and Medication of ESRD Subjects With and Without Renal Transplant in the Propensity Score-Matched Cohorts

The overall incidence rate of septicemia was approximately 1.3 times higher in the non-RTx group than in the RTx group (Table 2). Compared to the non-RTx group, the overall HR of septicemia in the RTx group was 0.73 (95% CI = 0.64–0.84, *P* < 0.001). The relative beneficial effect associated with transplantation was greater for young agers (HR: 0.64, 95% CI = 0.52–0.80, *P* < 0.001), males (HR: 0.68, 95% CI = 0.56–0.82, *P* < 0.001), those with a CCI score of ≥3 (HR: 0.47, 95% CI = 0.29–0.76, *P* < 0.01), and those used immunosupressants (HR: 0.61, 95% CI = 0.40–0.94, *P* < 0.05). The beneficial effect was not significant for patients with comorbidities of hepatitis B or patients took steroids. RTx recipients with hepatitis C were at higher risk of septicemia, but not significant. The analysis of interaction between factors showed that RTx status was significantly interacted with CCI (*P* = 0.003) and with hepatitis C (0.002). The incidence of septicemia in the RTx group was higher in the first 3 months follow-up period. It decreased to a level of 37% lower than the incidence in the non-RTx comparison group (1.85 vs. 2.73 per 100 person-years) (*P* < 0.001) after 12-month follow-up.

**TABLE 2:**
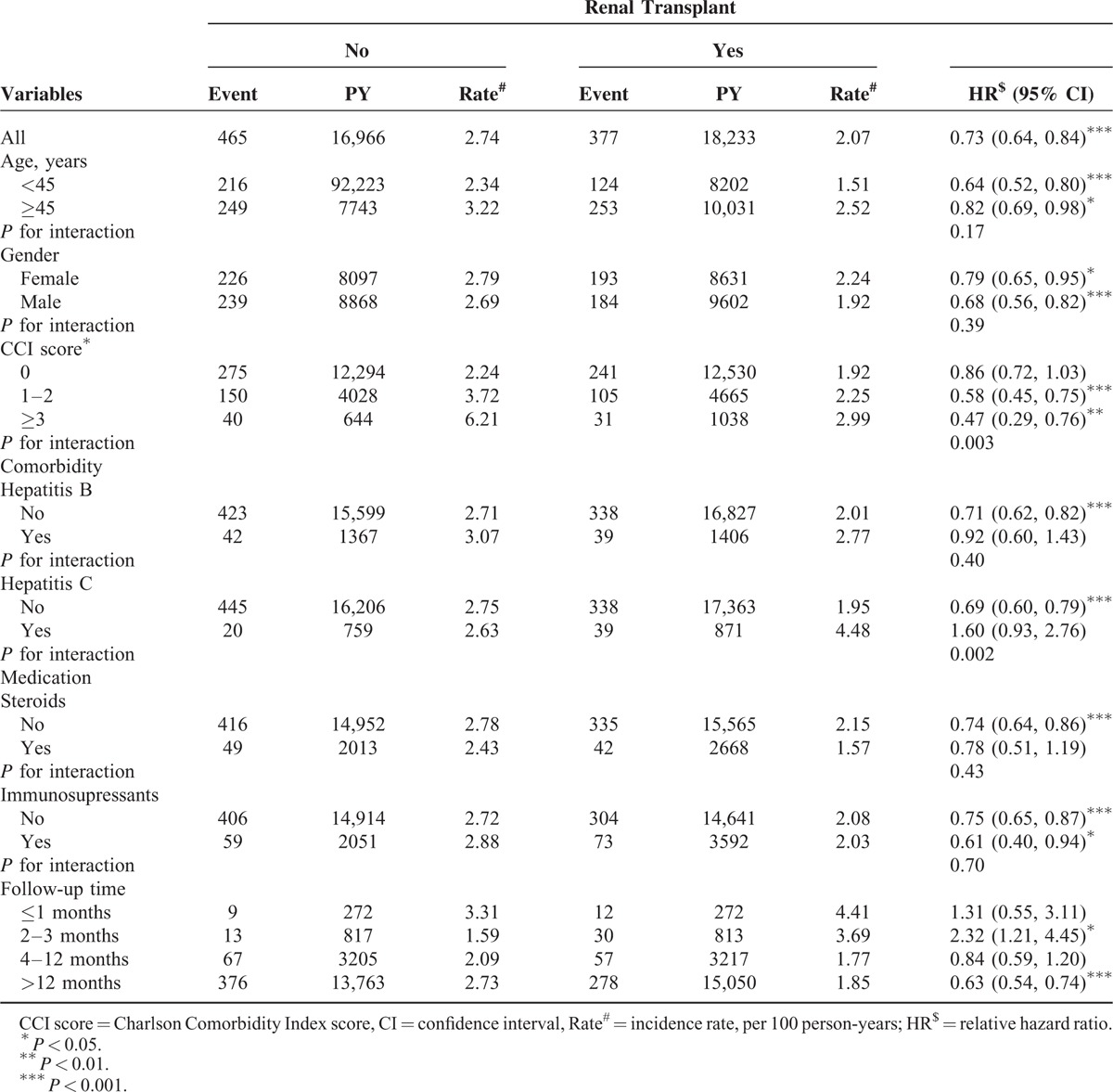
Septicemia Incidence in ESRD Subjects With and Without Renal Transplant and Cox Proportional Hazards Regression Analysis Estimated Relative Hazard Ratio

**FIGURE 2:**
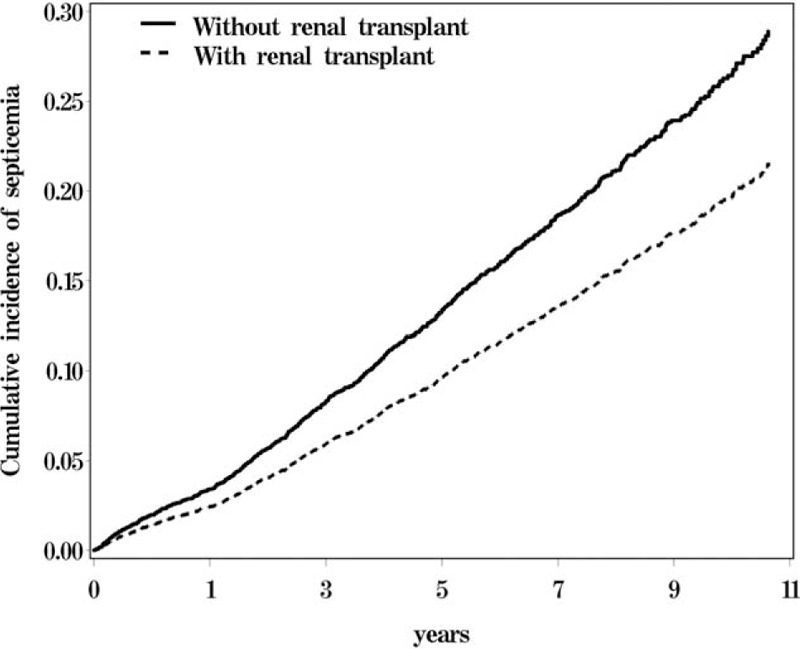
Cumulative incidence of septicemia estimated for ESRD patients with and without renal transplant.

The cumulative incidence of septicemia was 6.2% lower in the RTx group than in the non-RTx group (22.1% vs. 28.3%, *P* < 0.001) (Figure 2). Further data analysis showed that the incidence of repeat septicemia was also lower in the RTx group than in the non-RTx group (0.36 vs. 0.55 per 1000 person-years; or n = 66 vs. 93) with a HR of 0.63 (95% CI = 0.46–0.86) (data not shown).

Compared to patients in the non-RTx group, RTx recipients had a lower rate of emergency care uses (35.8% vs. 39.8%, *P* = 0.24) for septicemia and a lower all-causes mortality (17.2% vs. 18.5%, *P* = 0.64) since the occurrence of septicemia, but not significant (Table 3). However, further data analysis showed that the mean cost for the care of septicemia was greater for the RTx group (7175, SD = 14,283 in USD) than for the non-RTx group (6421, SD = 11,105 in USD) (data not shown). The mean cost of ICU cares was also greater for the RTx group than for the non-RTx group (14,552, SD = 20,514 vs. 11,390, SD = 15,638 in USD).

**TABLE 3:**
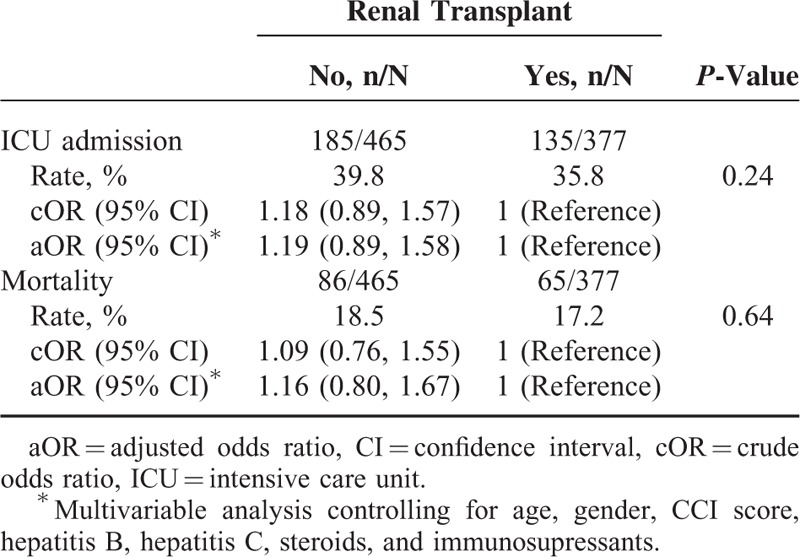
Odds Ratios of Intensive Care Unit Admission for Septicemia and 30-Day All-Cause Mortality Since Septicemia Diagnosed in the Propensity Score-Matched Cohorts

## DISCUSSION

To the best of our knowledge, this is the first study to evaluate the subsequent risk of septicemia for ESRD patients who have received RTx, using a propensity-matched method. The key finding of this study was that the incidence of septicemia among the RTx recipients was significantly lower than the propensity-matched ESRD patients. We considered this is related to the improvement of renal function and host defense and maintenance of skin and mucosa barriers, absence of invasive dialysis procedures, and decreased nosocomial transmission. The same reasons may also explain why the protective effectiveness is even greater for RTx patients with increasing CCI scores. RTx patients are benefited with a good amount of reduced septicemia risk for those with a high CCI score. This phenomenon has not been well reported in the literature. Our findings are also compatible with the well-known concept that the incidence of septicemia is higher in older patients and those with more comorbidities, which was observed in both groups.

Our study demonstrated that the HRs of septicemia in the RTx group compared with the non-RTx group were not consistent in the follow-up period. There was a decreasing trend of incident septicemia in the RTx group. The possible explanation is that operation-related complications may initially increase incident septicemia in the RTx group.^15^ Our further data analysis showed that the RTx group had higher incident pneumonia in the first month of follow-up, but not significant. The induction therapy of immunosuppression may also contribute to the increased incidence of septicemia soon after the transplantation and the risk declines with time. On the other hand, the risk of septicemia in the non-RTx group was in an increasing trend to a level higher than that in the RTx group.

In a recent study, Kalil et al^16^ reported that 28- and 90-day mortalities were significantly decreased for solid organ transplant recipients compared with nontransplant patients with bacteremic sepsis (adjusted HR: 0.22, 95% CI = 0.09–0.54, *P* = 0.001 and adjusted HR: 0.43, 95% CI = 0.20–0.89, *P* = 0.025). The authors suggested that the immunosuppression in transplant recipients may provide a survival advantage to those with sepsis through modulation of the inflammatory response. However, this viewpoint remains controversial. In the present study, we noticed the benefit from incident septicemia in the RTx group compared with the non-RTx group, but the ICU admission and 30-day all-cause mortality rates were insignificantly lower in the RTx group.

The annual report of United States Renal Data System (USRDS) in 2010 showed that peritoneal dialysis patients had the highest rate of admission for any infection (558 per 1000 patient-years), followed by hemodialysis patients (460 per 1000 patient-years) and RTx recipients (210 per 1000 patient-years).^17^ The corresponding rates of admission for bacteremia/sepsis in these 3 groups of patients were 116 versus 80 and 50 per 1000 patient-years, respectively. In the present study, we performed a precise age-, sex-, and comorbidity-matched method for establishing the comparison group, and the results were in accordance with these data.

Infection is one of the most common causes of post-RTx rehospitalization.^18–21^ The duration of post-RTx rehospitalization may be linked to the cause of admission. Naderi et al^22^ reported that the median stay of post-RTx rehospitalization was 5 days for nephrolithiasis, 7 days for surgical complications, 8 days for malignancy, 9 days for infection, and 10 days for renal dysfunction. Rehospitalization for infection correlated with a higher risk of prolonged stay. The average costs of such admissions sometimes equal or surpass the costs of the transplantation itself.^23^ In the present study, the overall mean cost for treating septicemia was higher in the RTx group than in the non-RTx group. The costs may include the expense of immunosuppressants in our study. In any case, the prolonged hospitalization, extra examinations, and advanced medications may contribute for the most part to this condition.

For the study design, we selected “septicemia” as the primary outcome, which has been previously used in other study.^16^ Septicemia is a more definitive diagnosis and should be diagnosed with a blood-culture-proven pathogen. It is a severe and disseminated type of infection with a high mortality rate. However, septicemia could include not only blood stream infection but any sites of advanced infection such as pneumonia, urinary tract infection, skin, and soft tissue infection. In contrast, viral infections, such as cytomegalovirus (CMV) and BK virus, which are well known to be prevalent in RTx recipients, might not be shown in the present study.

With respect to the validity of diagnosis, ESRD is categorized as a “catastrophic illness” and patients with ESRD requiring long-term renal replacement therapy are entitled to apply for the “catastrophic illness certificate” issued by the Taiwan insurance authority. The catastrophic illness-certified patients are eligible for a considerable discount with regard to medical expenses. The certification process requires critical evaluation of medical records and/or pathological reports by physicians specialized in the disease field.^24^ Septicemia is a relatively definitive diagnosis; therefore, NHIRD provides a reliable data source for ESRD and septicemia.

The strength of this study is in providing a large-scale, population-based, propensity-matched evaluation of ESRD patients with or without RTx and the subsequent risk of developing septicemia. A cohort study using insurance or register data is an economical method. However, there are several limitations to be considered when interpreting the present findings. First, the present study identified diseases using the ICD-9-CM algorithm from the claims data, rather than clinical diagnoses. These large administrative databases may be misleading or inexact. To avoid coding errors, we identified only disorders with repeated cares for this study. Septicemia, hepatitis B, and hepatitis C are serious disorders and are less likely to be mistakenly coded. The comorbidity with only one diagnosis was not considered valid. The Taiwan NHI program classifies ESRD as a catastrophic illness, which requires the insurance system to undergo a rigid review to register a patient for the care as catastrophic illness. Second, NHIRD does not provide detailed information on the primary causes of ESRD, environmental factors, occupation, smoking habits, alcohol consumption, body mass index, diet preference, or family history although these may be potential confounding factors. In addition, relevant clinical variables, such as renal function tests, serum laboratory data, imaging results, or culture reports, were unavailable in the study.

## CONCLUSION

RTx recipients had a significantly reduced risk of developing septicemia compared to the propensity-matched ESRD patients without RTx. This beneficial effect is of particularly important for patients with high CCI scores. RTx may also reduce the septicemia-associated ICU admission and 30-day all-cause mortality slightly but without statistical significance.
